# The impact of systemic lupus erythematosus on the risk of infection after total hip arthroplasty: a nationwide population-based matched cohort study

**DOI:** 10.1186/s13075-020-02300-1

**Published:** 2020-09-14

**Authors:** Chien-Hao Chen, Tien-Hsing Chen, Yu-Sheng Lin, Dave W. Chen, Chi-Chin Sun, Liang-Tseng Kuo, Shih-Chieh Shao

**Affiliations:** 1 Department of Orthopedic Surgery, Chang Gung Memorial Hospital, Keelung, Taiwan; 2 Division of Cardiology, Department of Internal Medicine, Chang Gung Memorial Hospital, Chang Gung University College of Medicine, Keelung, Taiwan; 3 Biostatistical Consultation Center of Chang Gung Memorial Hospital, Keelung, Taiwan; 4 Division of Cardiology, Department of Medicine, Chang Gung Memorial Hospital, Chiayi, Taiwan; 5 Department of Ophthalmology, Chang Gung Memorial Hospital, Keelung, Taiwan; 6 Department of Chinese Medicine, College of Medicine, Chang Gung University, Taoyuan, Taiwan; 7 Department of Medical Research and Development, Chang Gung Memorial Hospital, Keelung, Taiwan; 8 Division of Sports Medicine, Department of Orthopedic Surgery, Chang Gung Memorial Hospital, Chiayi, Taiwan. No. 6 West Sec, Chia-Pu Road, Putz City, Chiayi, Taiwan; 9 Department of Medicine, College of Medicine, Chang Gung University, Taoyuan, Taiwan; 10 Department of Pharmacy, Keelung Chang Gung Memorial Hospital, Keelung, Taiwan; 11 School of Pharmacy, Institute of Clinical Pharmacy and Pharmaceutical Sciences, College of Medicine, National Cheng Kung University, Tainan, Taiwan

**Keywords:** Systemic lupus erythematosus, Total hip arthroplasty, Periprosthetic joint infection

## Abstract

**Background:**

We aimed to assess the impact of systemic lupus erythematosus (SLE) on the risk of infection after total hip arthroplasty (THA).

**Methods:**

We identified patients undergoing primary THA (1996–2013) in Taiwan National Health Insurance Research Database (NHIRD). Patients were then divided into the SLE and control groups according to the diagnosis of SLE. We used 1:1 propensity score to match the control to the SLE group by age, sex, and comorbidities. The primary outcome was infection, including early and late superficial wound infection and periprosthetic joint infection (PJI). The secondary outcome was in-hospital complications.

**Results:**

We enrolled 325 patients in each group. In the primary outcome, the incidence of early superficial wound infection and PJI was comparable between the SLE and matched-control group. However, the incidence of late superficial wound infection and PJI in the SLE group was higher than that in matched-control group (11.4% vs. 5.5%, *P* = 0.01; 5.2% vs 2.2%, *P* = 0.04, respectively). Furthermore, the SLE group had a higher risk for late superficial wound infection and PJI (hazard ratio = 2.37, 95% confidence interval (CI) 1.35–4.16; HR = 2.74, 95% CI 1.14–6.64, respectively) than the matched-control. Complications other than infection and in-hospital mortality cannot be compared because of very low incidence.

**Conclusions:**

SLE is a risk factor for developing late superficial wound infection and PJI, but not for early postoperative complications following THA. Clinical presentations should be monitored to avoid misdiagnosis of PJI in SLE patients after THA.

## Background

Systemic lupus erythematosus (SLE), as a complex autoimmune disease, can be associated with several complications caused by its disease course or adverse effect of treatments. Osteonecrosis (ON) is one of the common musculoskeletal complications following treatment of patients with SLE, developed in approximately 10.8% of patients with SLE [1]. Corticosteroid use is a major causative agent of ON [2]. Other immunosuppressant agents may also put patients with SLE on an elevated risk of developing ON [1]. Osteonecrosis of the femoral head (ONFH) may result in groin pain, disability, and lower quality of life. Total hip arthroplasty (THA) performed on patients with SLE with ONFH has previously been reported to be an acceptable treatment to achieve functional improvement [3–5]. During 1999–2005, the arthroplasty rate was increased among patients with SLE [3], and more patients underwent THA due to osteoarthritis instead of ONFH because of improved survival. A cohort study reported that long-term survival of Chinese patients with SLE was comparable to that of Caucasian patients in the 1990s [6]. Thus, these patients live longer until joint degeneration develops.

The major concern about patients with SLE undergoing joint replacement was infection risk. Periprosthetic joint infection (PJI), a catastrophic complication following joint replacement, may result in sepsis, requiring repeated debridement and even removal of the prosthesis and subsequent significant disability and mortality. PJI could be classified as early and late based on the time of onset. Early PJI was defined as occurrence within 3 months after the index date of joint replacement. The most common etiology was contamination during the THA procedure. Late PJI was defined as occurrence after 3 months following the index surgery [7]. It can be related to direct invasion of bacteria from the wound or hematogenous infection from other infections, such as urinary tract infection or infected gallbladder. In previous studies, the outcome of patients with SLE after undergoing THA was conflicting. SLE and the effect of steroid or immunosuppressant agents on joint replacement arthroplasty were not clearly defined. Several studies reported increased complications or adverse events in patients with SLE undergoing arthroplasty [8–10]; however, other studies have reported contradictory results [11–13]. However, these studies only reported short-term surgical outcomes and did not focus on surgical site infection (SSI). Moreover, these studies were single-institutional designs with a limited sample size, which may limit the extrapolation of the study results.

The current study aimed to evaluate the impact of SLE on the risk of developing infection after THA, comparing with a matched cohort of those without SLE. We hypothesized that SLE is an independent risk factor for developing superficial wound infection or PJI after THA.

## Methods

### Data source

Our data were obtained from Taiwan National Health Insurance Research Database (NHIRD), which included data from 23 million individuals since 1995. This study was reviewed and approved by the institutional review board of Chang Gung Memorial Hospital (IRB CGMH 103-5040B).

### Study cohort enrollment and exclusion criteria

This is a retrospective cohort study. We used International Classification of Diseases, Ninth Revision, Clinical Modification (ICD9-CM) codes to identify patients who underwent THA from 1996 to 2013. Those who were admitted to the hospital and underwent primary THA for ONFH or OA during the study period were enrolled in the study cohort. These patients were further categorized based on the status of SLE, which was confirmed by the catastrophic illness certificate in NHIRD. Patients who were under 18 years of age, major trauma cases (ICD-9 959.99), those with a history of malignancy, and those who received joint replacement other than THA before or during the study period were excluded. The control cohort was sampled from the NHIRD, with the same inclusion and exclusion criteria, but excluding patients with SLE.

### Outcomes measurement

The primary outcome was infection, including early superficial wound infection (< 3 months from index surgery), early PJI, late (> 3 months from index surgery) superficial wound infection, and late PJI. The timing of 3 months was based on the definition of the Center for Disease Control (CDC) for PJI [7]. The definition of superficial wound infection was defined as SSI requiring further admission to hospital after index procedure with surgical procedures not involving the joint cavity (e.g., debridement) or without surgical procedures (e.g., cellulitis or mild wound infection). If the surgical procedures involved the prosthesis or joint cavity (e.g., prosthesis removal, arthrotomy, synovectomy, or Girdlestone procedure), these were defined as PJI [14]. The follow-up time was until the patient’s death or until the end of the NHIRD data recording. The secondary outcome was in-hospital complications, including pneumonia, urinary tract infection, pulmonary embolism, sepsis, deep vein thrombosis, and in-hospital mortality. All the events were detected by the ICD-9 CM coding in the following admissions (Additional file 1).

### Statistical analysis and propensity score matching

The distribution of demographic characteristics and in-hospital complications between the SLE and control groups were compared using Student’s *t* test and the chi-square test. We identified the comorbidities of both groups, including coronary arterial disease (CAD), diabetes mellitus (DM), chronic obstructive pulmonary disease (COPD), chronic kidney disease (CKD), cirrhosis, congestive heart failure (CHF), cerebral vascular accidents (CVA), and end-stage renal disease (ESRD).

First, we used the 1:1 propensity score to match the control group to the SLE group by age, sex, and comorbidities because the differences between these factors were substantial (Table 1). These confounding factors may affect the outcome of risk in developing PJI or complications after joint arthroplasty. The CCI (Charlson comorbidity index) score was excluded in the factor in performing propensity score matching because connective tissue disease is a component of the CCI score, wherein patients with SLE will be assigned one more score compared with the control group. Each patient in the SLE group was matched with one counterpart in the non-SLE group to achieve minimal bias. An absolute standardized mean difference of < 0.1 between the two groups after propensity score matching was considered well balanced. Second, Cox proportional hazards model was performed. Results were expressed as hazard ratios (HRs) and their 95% confidence intervals (CIs). Analyses were conducted using SAS software, version 9.4 (SAS Institute Inc., Cary, NC, USA).

**Table 1:** Baseline characteristics of patients who underwent THA before and after 1:1 propensity score matching^a^

	Before match			After match		
	SLE	Control	*P* value	SLE	Control	*P* value
	(*n* = 617)	(*n* = 2414)		(*n* = 325)	(*n* = 325)	
Age	38.7 ± 12.7	56.7 ± 14.5	< 0.01	44.3 ± 12.0	46.1 ± 14.0	0.07
Female	503 (81.5%)	999 (41.4%)	< 0.01	237 (72.9%)	237 (72.9%)	1.00
Comorbidities						
Cerebral vascular accidents	39 (6.3%)	247 (10.2%)	< 0.01	27 (8.3%)	17 (5.2%)	0.11
Coronary heart disease	46 (7.5%)	412 (17.1%)	< 0.01	45 (13.8%)	35 (10.7%)	0.09
Congestive heart failure	38 (6.2%)	120 (5%)	0.26	19 (5.8%)	11 (3.4%)	0.13
Chronic obstructive pulmonary disease	59 (9.6%)	320 (13.3%)	< 0.01	43 (13.2%)	35 (10.8%)	0.11
Diabetes mellitus	28 (4.5%)	450 (18.6%)	< 0.01	27 (8.3%)	22 (6.8%)	0.45
Cirrhosis	7 (1.1%)	122 (5.1%)	< 0.01	3 (0.9%)	5 (1.5%)	0.48
Chronic kidney disease	87 (14.1%)	71 (2.9%)	< 0.01	29 (8.9%)	20 (6.2%)	0.10
Dialysis	30 (4.9%)	13 (0.5%)	< 0.01	9 (2.8%)	5 (1.5%)	0.28
CCI_Score	2.49 ± 1.51	1.58 ± 1.78	< 0.01	2.37 ± 1.42	1.36 ± 1.71	< 0.01

*CCI* Charlson comorbidity index, *SLE* systemic lupus erythematosus

^a^Data are presented as number and percentage or mean ± standard deviation

### SLE disease activity and 5-year infection rate after THA

To further investigate the disease activity of SLE patients and its effect on the risk of infection after receiving THA, we analyzed the outpatient and inpatient medication 3 months before the index THA surgery using the Anatomical Therapeutic Chemical (ATC) classification system. The medication included oral or parenteral glucocorticoid, conventional disease-modifying antirheumatic drugs (DMARDs) (hydroxychloroquine, azathioprine, mycophenolate mofetil, ciclosporin, cyclophosphamide, methotrexate), and biological DMARD (Rituximab). We defined the infection outcome as any superficial infection or PJI within 5 years after the index surgery. We used Student’s *t* test, the chi-square test, and Fisher’s exact test to compare the exposure of medication and how many kinds of DMARDs were used in infection and non-infection cases. Meanwhile, we further recorded the 5-year infection rate by year at which the index surgery was started and analyzed the trend of infection rate during the study period.

## Results

**Fig. 1:**
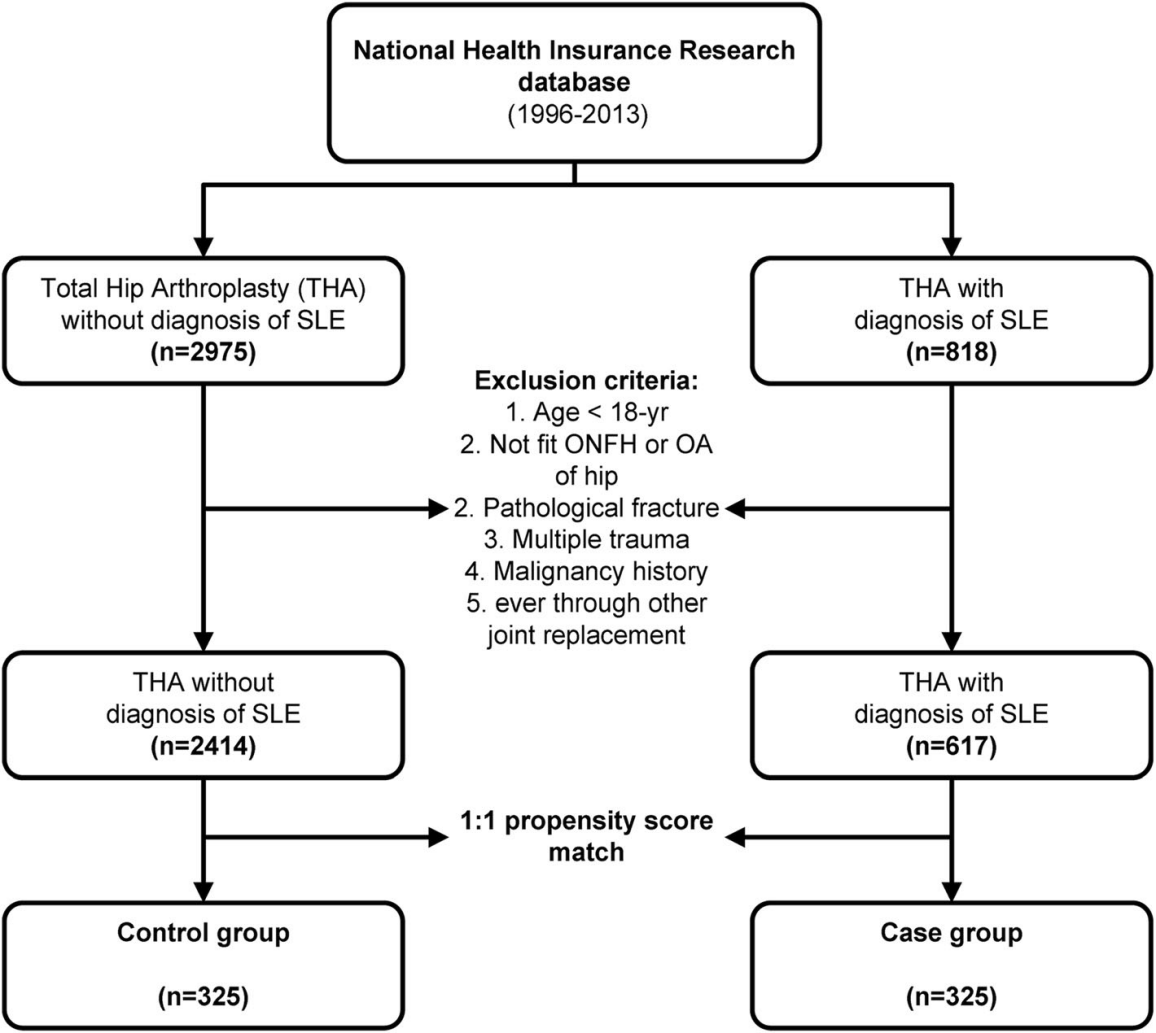
Flow chart of study cohort selection. ONFH = osteonecrosis of femoral head; SLE = systemic lupus erythematosus; THA = total hip arthroplasty

We identified 818 patients with SLE who underwent THA from 1996 to 2013. In the million sampling of NHIRD, we identified 2975 patients without SLE who underwent THA during the study period. After applying the exclusion criteria, 617 THA patients with SLE (SLE group) and 2414 patients without SLE (control group) remained. After 1:1 propensity score matching, 325 patients were enrolled in both SLE and matched-control groups (Fig. 1).

### Patient’s characteristics

Before matching, the SLE group was significantly younger than the control group (mean age: 38.7 vs. 56.7, *P* <  0.01) and female predominant (81.5% vs. 41.4%, *P* <  0.01). The SLE group had a higher rate of CKD or ESRD. Otherwise, the control group had significant a higher rate of comorbidities of CAD, DM, COPD, cirrhosis, and CVA. After matching, the age, sex, and comorbidities were comparable in both groups (Table 1). Concerning the etiology of receiving THA in SLE group, 88% (288 in 325) had a diagnosis of ONFH, 11% (36 in 325) had a diagnosis of OA, and the remaining one case were femoral neck fracture. In the control group, 41.5% patients (135 in 325) had a diagnosis of ONFH, 57.8% (188 in 325) had a diagnosis of OA, and the remaining two cases were femoral neck fracture. In each subgroup of ONFH or OA in SLE patients, the PJI rate was about 6–7%. In the control group, the PJI rate was about 3% in the ONFH and OA subgroups (Table S1). The diagnosis of ONFH or OA did not significantly affect the risk of developing PJI after THA in SLE and control groups.

### Infection

The incidence of early superficial wound infection rate was not significantly different between the SLE and the matched-control groups (2.2% vs. 0.6%, *P* = 0.9, Table 2). The incidence of early PJI was not different between the two groups (1.5% vs. 0.6%, *P* = 0.25, Table 2). However, the incidence of late superficial wound infection and late PJI was higher in the SLE group (11.4% vs. 5.5%, *P* = 0.01; 5.2% vs. 2.2%, *P* = 0.04, respectively). Using the Cox regression model, the SLE group had a higher risk of developing late superficial wound infection (HR 2.37; 95% CI, 1.35–4.16, Table 2) and late PJI (HR 2.74; 95% CI, 1.14–6.64, Table 2).

**Table 2:** Infection between SLE and matched-control patients^a^

	SLE	Control		SLE vs. control	
	(*n* = 325)	(*n* = 325)	*P*	HR	95% CI
Early infection (< 3 months)					
Early superficial wound infection	7 (2.2%)	2 (0.6%)	0.09	3.53	0.73–17.0
Early PJI	5 (1.5%)	2 (0.6%)	0.25	2.52	0.49–13.0
Late infection (> 3 months)					
Late superficial wound infection	37 (11.4%)	18 (5.5%)	0.01	2.37	1.35–4.16
Late PJI	17 (5.2%)	7 (2.2%)	0.04	2.74	1.14–6.64

*CI* confidence interval, *HR* hazard ratio, *PJI* periprosthetic joint infection, *SLE* systemic lupus erythematosus

^a^Data are presented as number and percentage

**Fig. 2:**
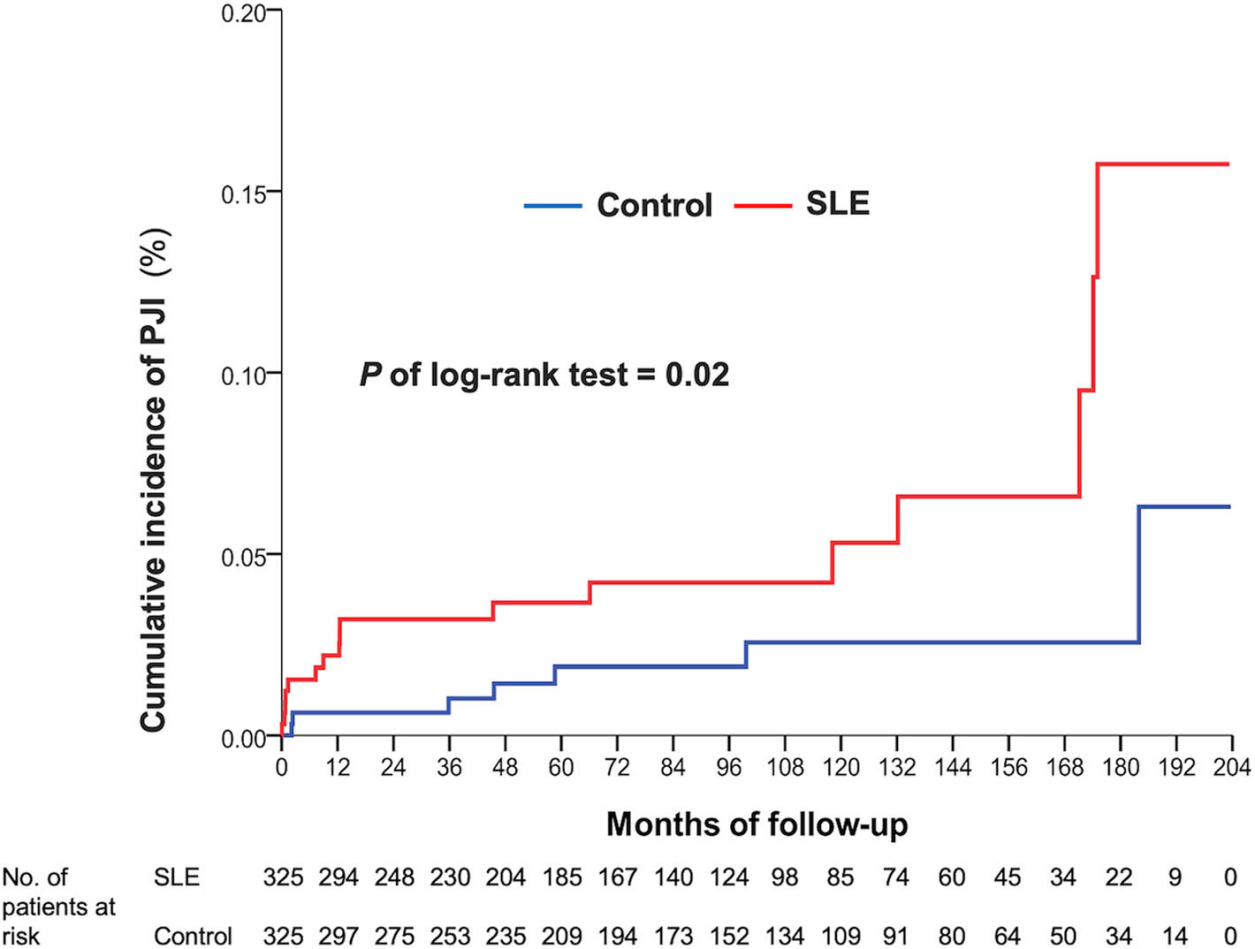
Cumulative incidence of prosthetic joint infection in SLE and matched-control group over a 17-year period

**Fig. 3:**
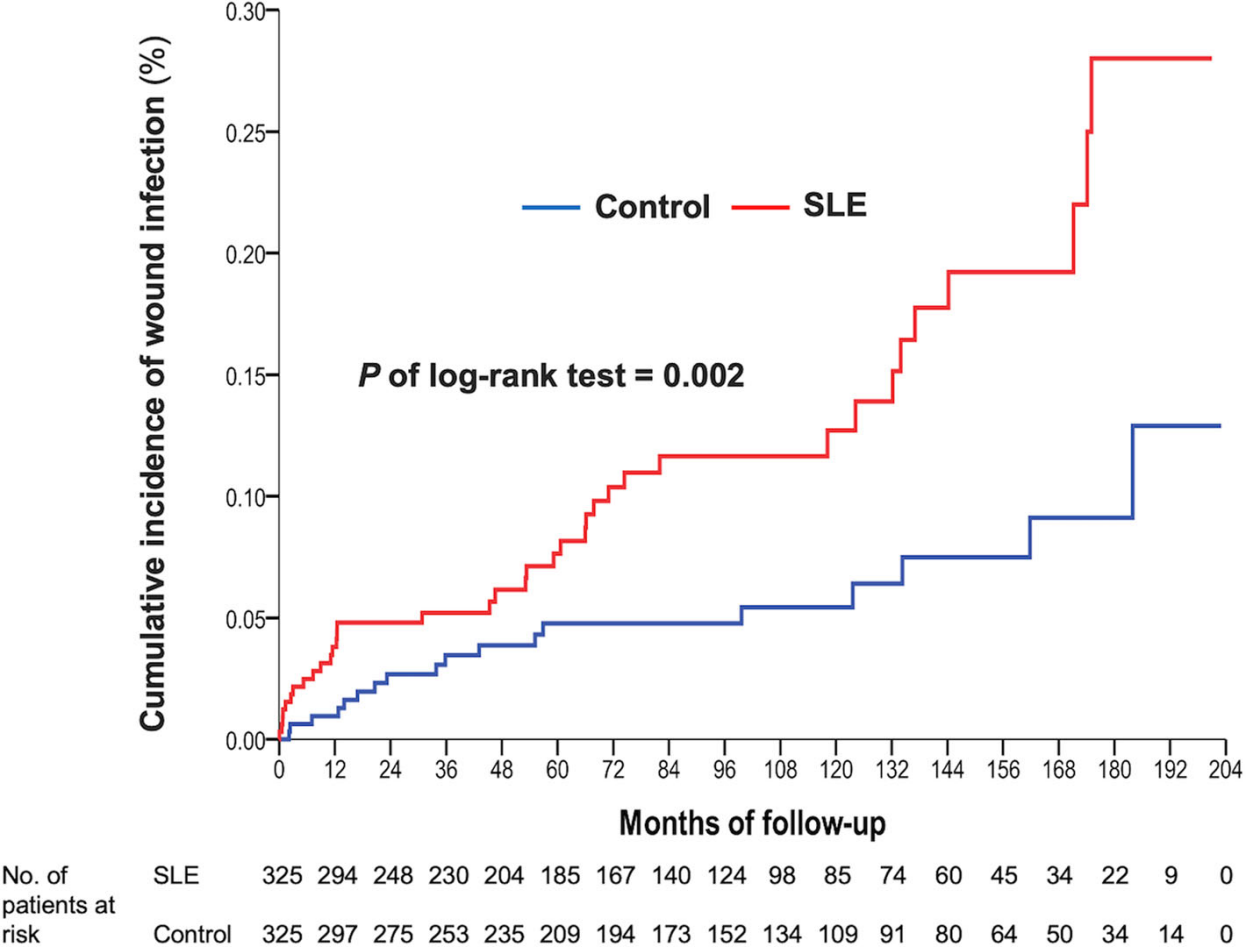
Cumulative incidence of wound infection in SLE and matched-control group over a 17-year period

Using the Kaplan–Meier method to compare the cumulative risk of infection in both groups, we found that the SLE group had a higher PJI or wound infection rate (*P* of log-rank test = 0.02 and 0.002). We found that the SLE group developed PJI clustered in the first-year follow-up, and superficial wound infection clustered around the 1st year, 4th–6th year, and 10th–12th year (Fig. 2 and Fig. 3).

### In-hospital complications other than infection

The length of stay, approximately 8 days, was comparable between the two groups. The complications other than infection and in-hospital mortality rates cannot be compared in both groups because of a very low incidence (Table 3).

**Table 3:** In-hospital outcomes between SLE and matched-control patients*

	SLE	Control	*P*
	(*n* = 325)	(*n* = 325)	
Pneumonia	0 (0.0%)	0 (0.0%)	1.00
Urinary tract infection	6 (2.5%)	7 (2.6%)	0.94
Pulmonary embolism	0 (0.0%)	0 (0.0%)	1.00
Sepsis	0 (0.0%)	0 (0.0%)	1.00
Deep vein thrombosis	1 (0.3%)	0 (0.0%)	0.31
In-hospital mortality	0 (0.0%)	0 (0.0%)	1.00

*SLE* systemic lupus erythematosus

^*^Data are presented as number and percentage

### SLE disease activity and 5-year infection rate after THA

**Fig. 4:**
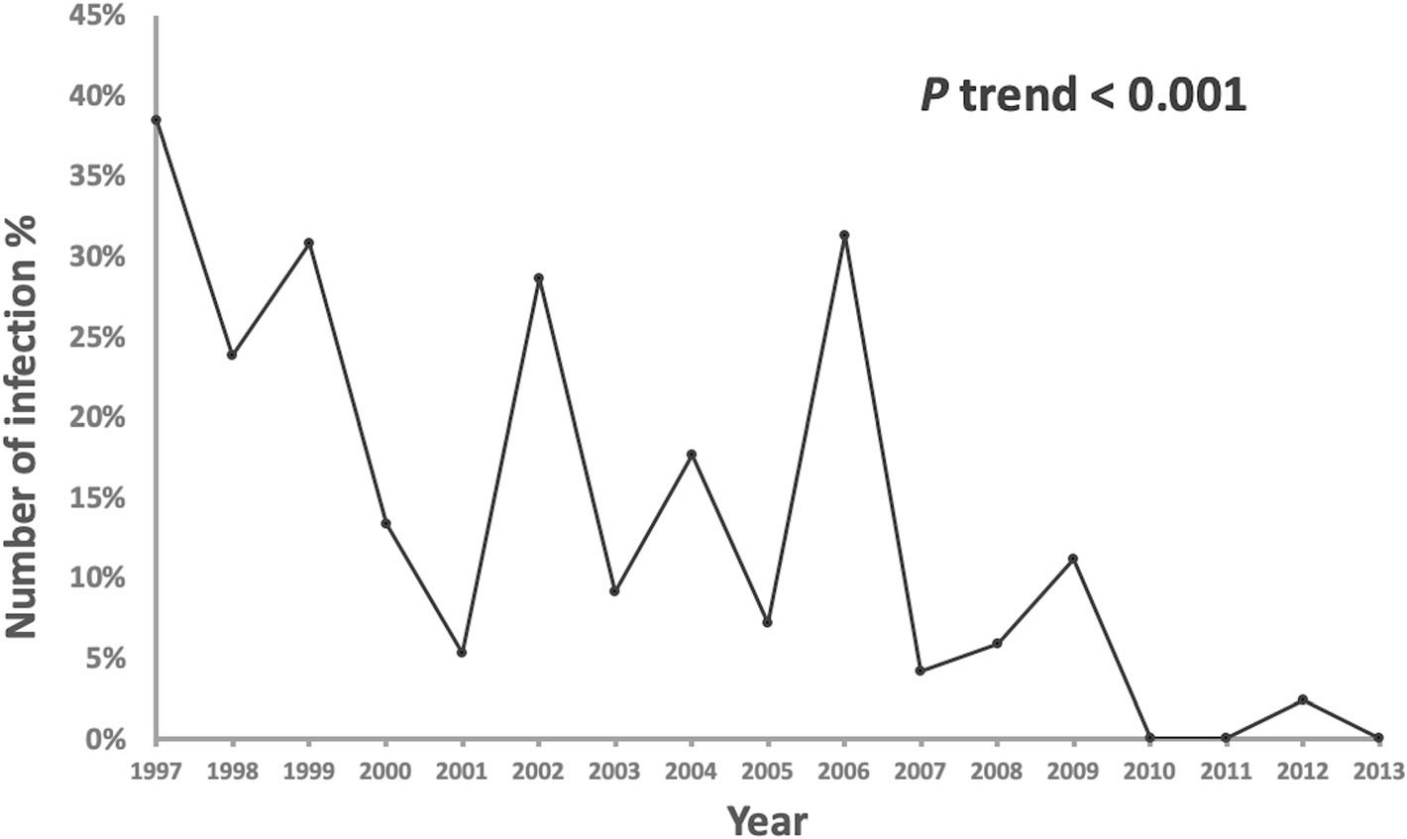
Five-year infection rate after THA in SLE patients during 1997–2013

A total of 37 cases developed infection with 5-year after index surgery. There was no significant difference between the infection and non-infectious cases in exposure of parenteral or oral steroid, hydroxychloroquine, cyclophosphamide, and methotrexate. But the azathioprine exposure in infection cases was significant less than non-infection cases (8.11% vs. 28.13%, *P =* 0.009, Table 4). Besides, the kinds of DMARDs used in infection cases were also significantly less than in non-infection cases (1.38 ± 0.76 vs. 1.81 ± 0.97, *P* = 0.002, Table 4). The patients receiving THA in the first half of the study period had a higher 5-year infection rate compared to those receiving THA in the second half of the study period (21.6% vs. 6.13%, *P* = 0.001, Table S2). The 5-year infection rate decreased significantly during the study period (*P* trend < 0.001) (Table S3 and Fig. 4).

**Table 4:** Medication exposure in SLE patients during 5-year follow-up after THA^a^

Medication	Infection cases (*n* = 37)	Non-infection cases (*n* = 288)	*P*
Steroid			
Parenteral	2 (5.41)	33 (11.46)	0.399
Oral	28 (75.68)	219 (76.04)	1
DMARDs-conventional			
Hydroxychloroquine	16 (43.24)	174 (60.42)	0.052
Azathioprine	3 (8.11)	81 (28.13)	0.009
Mycophenolate mofetil	0 (0.00)	9 (3.13)	–
Ciclosporin	0 (0.00)	6 (2.08)	–
Cyclophosphamide	2 (5.41)	10 (3.47)	0.634
Methotrexate	2 (5.41)	15 (5.21)	1
DMARDs-biological			
Rituximab	0 (0.00)	0 (0.00)	
DMARDs type	1.38 ± 0.76	1.81 ± 0.97	0.002

*DMARDs* disease-modifying antirheumatic drugs

^a^Data are presented as number and percentage

## Discussion

The principal finding of our study suggested that SLE would not significantly increase the risk of developing early superficial wound infection or PJI after receiving THA. But the risk of late superficial wound infection or PJI was significantly higher in patients with SLE, when comparing to those without SLE. The results implied that the advance of surgical technique and perioperative care makes patients with SLE to not have a higher risk of superficial wound infection or PJI in the early postoperative period after index THA surgery when compared with those without SLE. However, in a longer follow-up period, the immunosuppressive agent or the disease course would make patients with SLE more susceptible to superficial wound infection and PJI.

PJI, one of the most severe complications after total joint replacement, may significantly affect joint function, quality of life, implant survival, and patient survival [15, 16]. The host immune status is crucial in developing PJI after joint replacement [17]. Compared with OA, rheumatoid arthritis was previously reported as a risk factor for PJI [18]. The literature showed that the effect of SLE on the infection after THA is uncertain. A systematic review in 2015 had collected data of 162 patients with SLE who underwent 214 THA, with a mean follow-up of 72.5 months [19]. The mean Harris Hip Score improved from 45.5 preoperatively to 88.6 at the last follow-up. At least one complication was experienced by 17% of patients. Superficial wound infection occurred in 3.3% of participants. Kasturi et al. reported that pain and functional outcomes after arthroplasty in patients with SLE were comparable to those without SLE, but lupus remained an independent risk factor for post-hip arthroplasty complications and mortality [9]. Another case-control study compared the THA outcomes between patients with SLE and matched OA controls and showed that SLE was an independent risk factor for adverse events after THA [20]. Patients with SLE had higher rates of falls, acute renal disease, infections, and revision surgeries than the matched OA controls. However, a recent large series study comparing the outcomes between patients with and without undergoing THA reported that patients with SLE had an increased risk of both medical- and surgical-related complications [21]. Considering the complications of superficial wound infection and PJI, this study did not find a significant difference between patients with and without SLE [21]. To date, no literature focused on the infection of THA in the long-term follow-up.

In our study, we matched patients with and without SLE to eliminate confounding factors, such as age and comorbidities. We found that the disease status of SLE would not significantly increase the risk of perioperative wound infection or PJI. Besides, the risk of in-hospital complication and mortality was comparable between both groups. Because early superficial wound infection and PJI were mainly caused by intraoperative contamination of high-virulence bacteria, the findings of this study show that patients with SLE undergoing THA did not have an elevated risk for the development of major complications, including SSI. This may be accused that patients with SLE received THA at a relatively younger age (mean, 48 years) when compared with the general population. The immune function of these young patients with SLE was comparative to the control group. The latest CDC guideline for prevention of SSI reviewed and concluded that the effect of steroid and immunosuppressive agent on the occurrence of SSI in prosthesis joint arthroplasty was uncertain [22]. Although the use of steroid, biologic agent, and DMARDs before joint replacement arthroplasty should be carefully evaluated and adjusted based on the 2017 American College of Rheumatology/American Association of Hip and Knee Surgeons Guideline to lower the potential adverse events, such as wound healing or SSI [23]. With adequate discussion of rheumatologic specialists and careful selection of the disease-stable patients, THA will be a safe procedure in improving the quality of life of patients with SLE with hip arthrosis or ONFH.

One of the causes of late PJI is the contamination of low-virulence pathogen intraoperatively. The time of onset is approximately 2–3 months to 2 years after THA, with the presentation of prosthesis loosening and persisting joint pain [24]. Besides, hematogenous PJI due to hematogenous spread from another infection site can occur at any time after joint replacement surgery [25]. The presentation includes fever, joint pain, swelling, disability, sepsis, and sinus tract discharge. If the onset of joint symptoms was more than 1 month, it was defined as chronic PJI [26]. The management is very different between acute and chronic PJI. In acute PJI, the treatment is arthrotomy and debridement with antibiotic treatment, the success rate is high, and the impact on patient’s quality of life and cost is relatively low. In chronic PJI, surgical treatment included prosthesis removal, antibiotic cement prosthetic spacer insertion, and second-stage revision joint arthroplasty. Management of chronic PJI is still related to surgical morbidity and decreased success rate, especially in immunocompromised patients [27]. In our data, we observed that in the long-term follow-up (> 3 months), the incidence of late PJI was significantly higher in the SLE group. Meanwhile, PJI mostly occurred in the first 12 months following the index surgery. Because patients with SLE are more susceptible to major infection [28, 29], such as pneumonia or urinary tract infection, those patients with prosthetic hip joint may sustain the risk of hematogenous spread. Clinically, patients with SLE who underwent THA should be suspicious for PJI when they had acute hip joint pain and swelling following an infection episode and should be treated adequately, especially in the first year. Another cause of low-virulence pathogen intraoperative seeding may be another cause of a higher late PJI rate in the SLE group. These patients should be carefully observed for signs of prosthesis loosening or referred to an orthopedic surgeon for further examination.

Another finding of our study is that superficial wound infection following THA in the SLE group was significantly higher than that in the control group in the longer follow-up period. The events were clustered in the 1st year and during the 4th to 6th year after THA. Most of these patients were diagnosed with cellulitis in our study cohort. The possible cause of cellulitis was decubitus ulcer caused by bony prominence at the greater trochanter. Combined with cutaneous complication of steroid use in patients with SLE, the skin was thinning and more prone to develop cellulitis or pressure ulcer around the hip. Because one of the methods of PJI was direct spread from the adjacent soft tissue infection, we should pay attention to the skin lesion around the hip clinically.

In the current study, SLE patients who developed infection after THA had less kinds of DMARD medication used compared with those who did not have infection. This result could be explained that using different types of DMARDs provides better disease control in SLE patients. These patients may need a relatively lower dosage of steroid, and hence less immunosuppression, although the exposure of steroid was not significantly different between infection or non-infection SLE patients. Furthermore, we observed a significantly lower 5-year infection rate in the second half of study period comparing with the first half, which could be attributed to the better disease control of SLE patients with the evolution of DMARDs [30]. Besides, the improved surgical technique and perioperative care could be the other factors contributing to the decreased infection rate.

The strength of this study was the largest series of patients with SLE undergoing THA with long-term follow-up (> 10 years). We matched the SLE and control groups by underlying disease to eliminate their effect on immune function and evaluate the independent effect SLE on the surgical outcome of THA. We focused on the outcome of SSI, including early superficial wound infection, early PJI, late superficial wound infection, and late PJI. However, our study had several limitations. First, the study only included patients with SLE who were in relatively stable disease condition or had less comorbidity. Those who were too ill to undergo surgery would be excluded. The selection bias may lower the risk of infection and perioperative complication rate. Second, we used ICD-9 CM codes to define the occurrence of PJI due to the lack of laboratory data or culture data in the NHI database. We could not further analyze the influence of different pathogens and outcomes of different severities of infections in patients with SLE. Third, the severity of SLE might affect the outcomes following THA. We could not further classify the severity of SLE in the included patients due to the lack of data; however, we utilized the exposure of medication to represent disease status in the SLE patients. Fourth, we cannot differentiate the fixation methods of femoral component in our database and cannot further investigate its effect on infection after THA. Further studies comparing different fixation methods will be needed to clarify this issue.

## Conclusions

For patients undergoing THA for ONFH or OA, SLE did not increase the risk of early superficial wound infection or PJI under the appropriate patient selection and management of immunosuppressants during the short-term follow-up. However, in longer follow-up, SLE is still a risk factor for late superficial wound infection or PJI. We should closely monitor the symptoms and signs of prosthetic hip joint to avoid delay or misdiagnosis of PJI in patients with SLE.

## Supplementary information

**Additional file 1:** Appendix 1. ICD-9-CM codes used for diagnosis in the current study. **Table S1.** Periprosthetic joint infection incidence by diagnosis for receiving THA. **Table S2.** Five-year infection rate in SLE patients by different time periods. **Table S3.** Five-year infection rate in SLE patients after THA by years.

## Data Availability

The data that support the findings of this study are available from Taiwan National Health Insurance Database, but restrictions apply to the availability of these data, which were used under license for the current study, and so are not publicly available. Data are however available from the authors upon reasonable request and with permission of National Health Insurance Administration and the Ministry of Health and Welfare.

## Abbreviations

CCI: Charlson comorbidity index
OA: Osteoarthritis
ONFH: Osteonecrosis of femoral head
PJI: Periprosthetic joint infection
SLE: Systemic lupus erythematosus
THA: Total hip arthroplasty
