# Supplementary material for: The impact of systemic lupus erythematosus on the risk of infection after total hip arthroplasty: a nationwide population-based matched cohort study
Source: Arthritis Res Ther. 2020 Sep 14;22:214. doi: 10.1186/s13075-020-02300-1 (PMC7488693; doi:10.1186/s13075-020-02300-1)
Supplement: Supplementary file 1 — Additional file 1: Appendix 1. ICD-9-CM codes used for diagnosis in the current study. Table S1. Periprosthetic joint infection incidence by diagnosis for receiving THA. Table S2. Five-year infection rate in SLE patients by different time periods. Table S3. Five-year infection rate in SLE patients after THA by years. [file 13075_2020_2300_MOESM1_ESM.docx]

**Appendix 1** ICD-9-CM codes used for diagnosis in the current study

|  | Diagnosis | ICD-9 |
| --- | --- | --- |
| Operative diagnosis | Systemic lupus erythematosus | 7100 |
|  | Osteonecrosis of the femoral head | 733.42, 733.43, 7334, 73340, 73341, 733.49, 733.81 |
|  | Osteoarthrosis of the hip | 715.35, 715.25,715.15,715.95, 715.16, 715.21, 715.26, 715.31, 715.36, 715.89, 715.90, 715.96 |
| Comorbidity | Cerebral vascular accident | 430.xx-438.xx |
|  | Coronary heart disease | 430.xx-438.xx |
|  | Congestive heart failure | 428.xx |
|  | Chronic obstructive lung disease | 491.xx, 492.xx, 494.xx, 496.xx |
|  | Diabetes mellitus | 250.xx |
|  | Cirrhosis | 571.2, 571.5, 571.6, 572.2, 572.3, 572.4, 572.8, 573.0 |
|  | Chronic kidney disease | 585.xx, 586.xx |
| In-hospital complication | Pneumonia | 480.x-486.x |
|  | Urinary tract infection | 599.0, 595.0 |
|  | Pulmonary embolism | 415.1x |
|  | Sepsis | 038.X, 790.7x |
|  | Deep vein thrombosis | 453.x |
| Readmission diagnosis | Disruption of operation (surgical) wound | 998.3,998.31, 998.32 |
|  | Postoperative infection | 998.5,998.51,998.59 |
|  | Non-healing surgical wound | 998.83 |
|  | Cellulitis of the hip | 682.6 |
|  | Pyogenic(septic) arthritis of the hip | 711.05 |
|  | periprosthetic joint infection of the hip | 996.66 |

ICD-9-CM, International Classification of Diseases, Ninth Revision, Clinical Modification

**Table S1** Periprosthetic joint infection incidence by diagnosis for receiving THA

|  | SLE | | Control | |
| --- | --- | --- | --- | --- |
|  | Case | PJI | Case | PJI |
| ONFH | 288 | 20(6.9%) | 135 | 4 (3.0%) |
| OA | 36 | 2 (5.6%) | 188 | 5 (2.7%) |
| Fracture | 1 | 0 (0%) | 2 | 0 (0%) |
| Total | 325 | 22(6.8%) | 325 | 9(2.8%) |

OA = osteoarthritis; ONFH = osteonecrosis of femoral head; PJI = periprosthetic joint infection; SLE = systemic lupus erythematotuss; THA = total hip arthroplasty.

**Table S2** Five-year infection rate in SLE patients by different time periods

| Time periods | THA cases | Infection* | *P* |
| --- | --- | --- | --- |
| 1997-2005 | 125 | 27 (21.6%) | 0.001 |
| 2006-2013 | 163 | 10 (6.13%) |  |

* infection includes superificial wound infection and periprosthtic joint infection.

SLE = systemic lupus erythematosus; c

**Table S3** Five-year infection rate in SLE patients after THA by years

| THR year | Case number | Number of infections* | 5-year infection rate (%) |
| --- | --- | --- | --- |
| 1997 | 13 | 5 | 38.46% |
| 1998 | 21 | 5 | 23.81% |
| 1999 | 13 | 4 | 30.77% |
| 2000 | 15 | 2 | 13.33% |
| 2001 | 19 | 1 | 5.26% |
| 2002 | 14 | 4 | 28.57% |
| 2003 | 11 | 1 | 9.09% |
| 2004 | 17 | 3 | 17.65% |
| 2005 | 28 | 2 | 7.14% |
| 2006 | 16 | 5 | 31.25% |
| 2007 | 24 | 1 | 4.17% |
| 2008 | 17 | 1 | 5.88% |
| 2009 | 18 | 2 | 11.11% |
| 2010 | 23 | 0 | 0.00% |
| 2011 | 12 | 0 | 0.00% |
| 2012 | 42 | 1 | 2.38% |
| 2013 | 22 | 0 | 0.00% |
| Total | 325 | 37 | 11.3% |

* The number of infection events were assorted into the year of index surgery.

SLE = systemic lupus erythematosus; THA = total hip arthroplasty.
